# Supplementary material for: Hospitalization Trends for Acute Appendicitis in Spain, 1998 to 2017
Source: Int J Environ Res Public Health. 2021 Dec 2;18(23):12718. doi: 10.3390/ijerph182312718 (PMC8656947; doi:10.3390/ijerph182312718)
Supplement: Supplementary file 1 [file ijerph-18-12718-s001.zip › ijerph-1459311-supplementary.pdf]

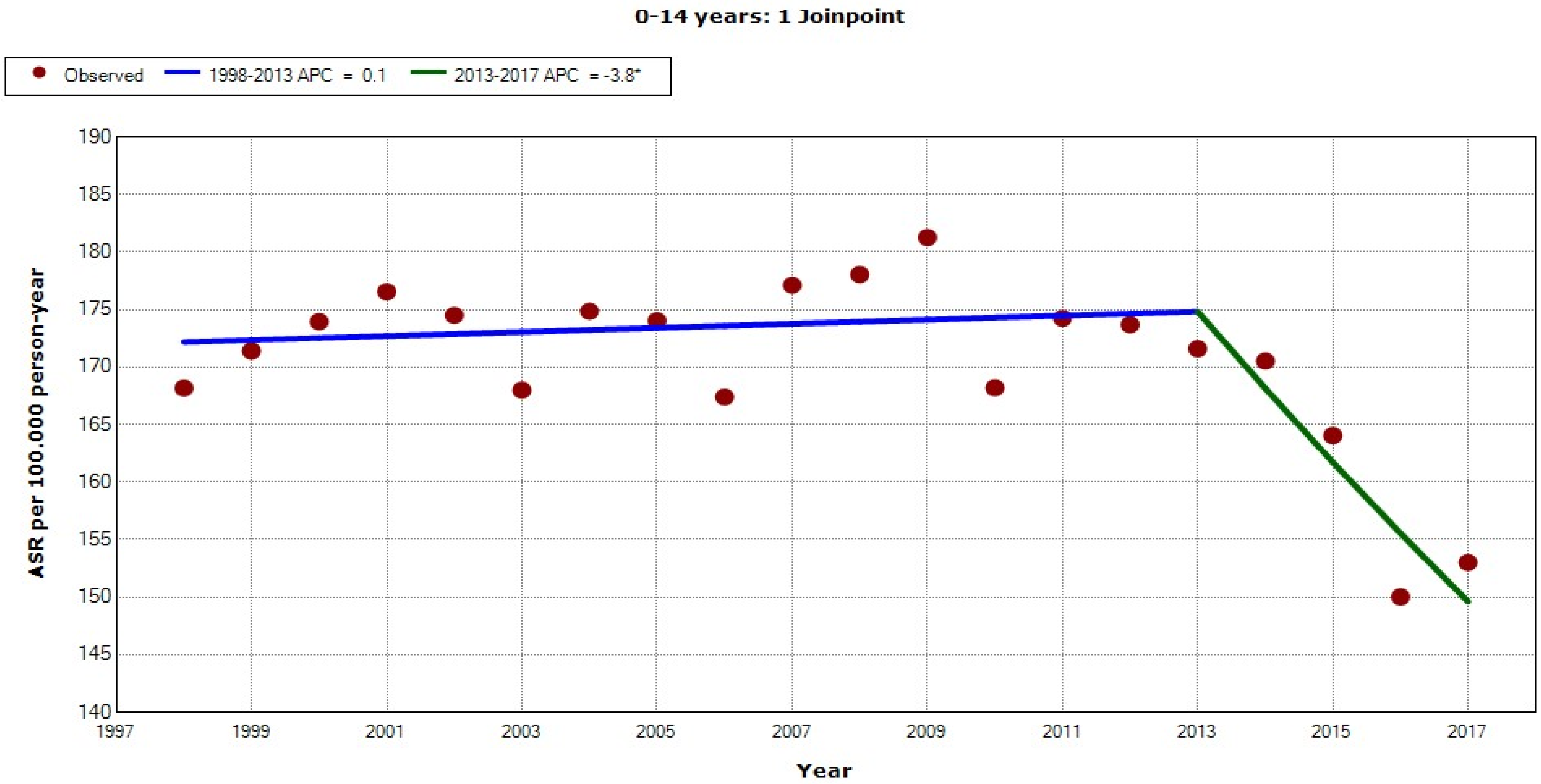

* Indicates that the Annual Percent Change (APC) is significantly different from zero at the alpha = 0.05 level.
Final Selected Model: 1 Joinpoint.

ARS: Annual Standardized rate

Supplementary Figure S1. Age-standardized rate of hospital admissions due to acute appendicitis per 100,000 population, in male aged 0 to 14 years in Spain, 1998 to 2017.

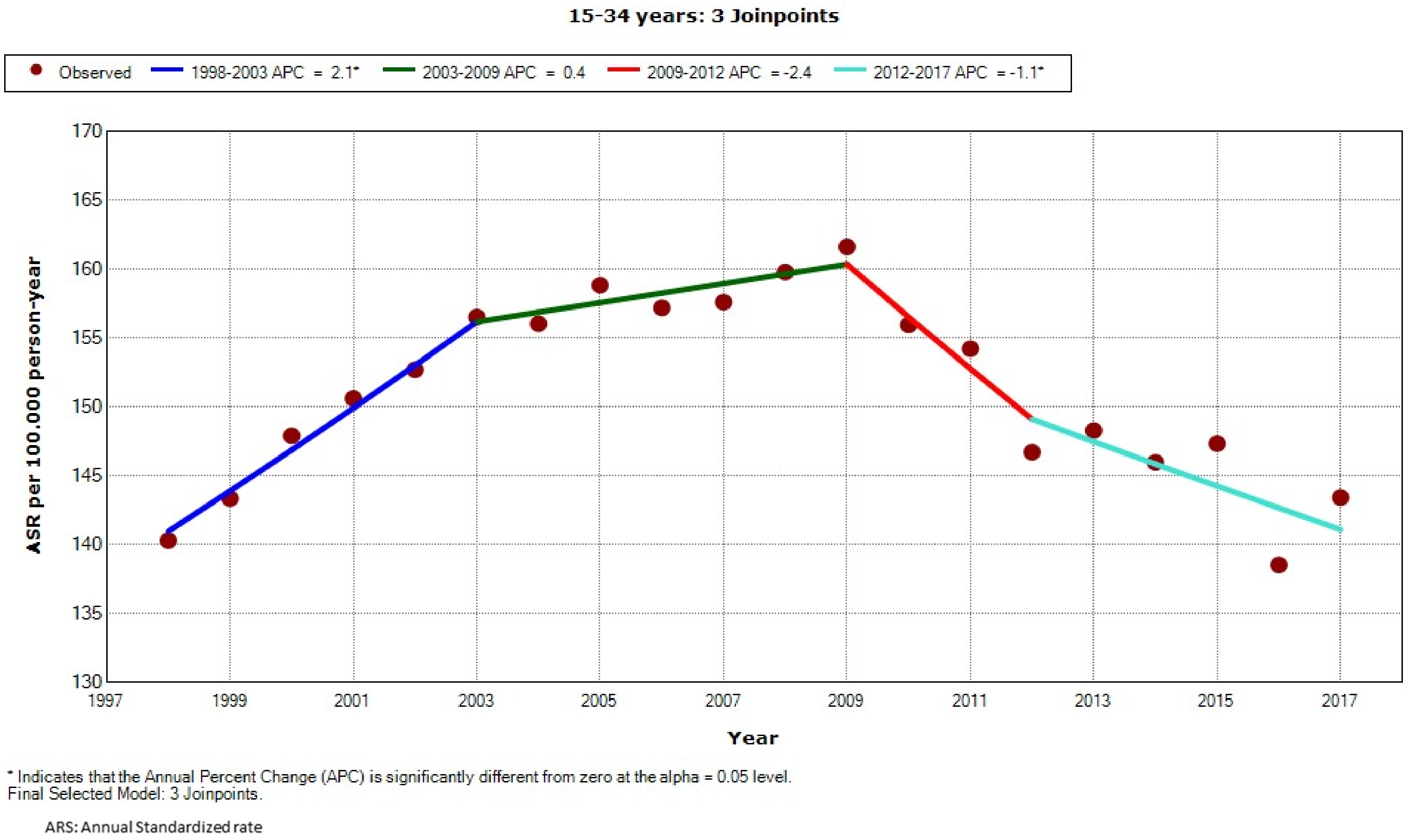

Supplementary Figure S2 Age-standardized rate of hospital admissions due to acute appendicitis per 100,000 population, in male aged 15 to 34 years in Spain, 1998 to 2017.

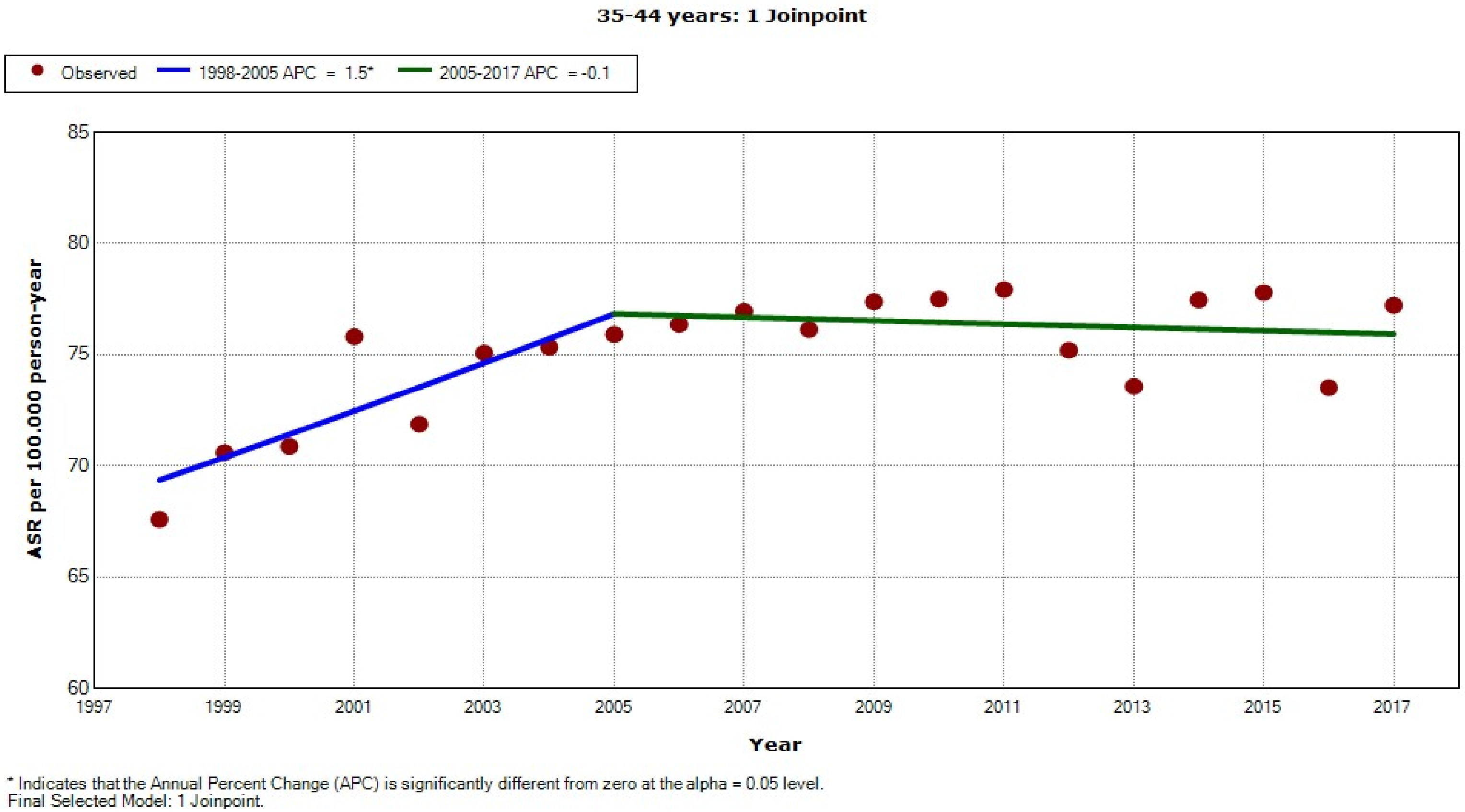

ARS: Annual Standardized rate

Supplementary Figure S3. Age-standardized rate of hospital admissions due to acute appendicitis per 100,000 population, in male aged 35 to 44 years in Spain, 1998 to 2017.

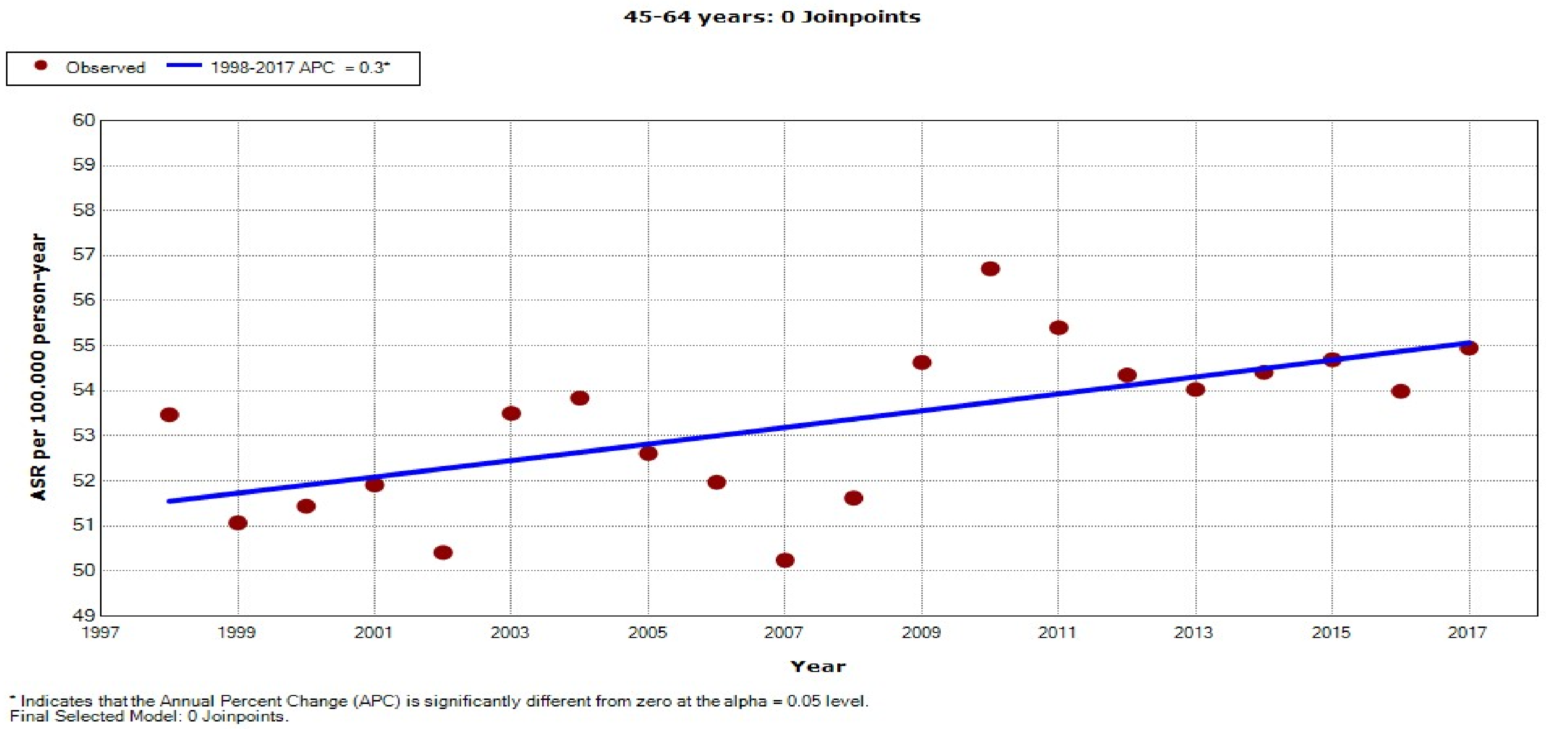

ARS: Annual Standardized rate

Supplementary Figure S4. Age-standardized rate of hospital admissions due to acute appendicitis per 100,000 population, in male aged 45 to 64 years in Spain, 1998 to 2017.

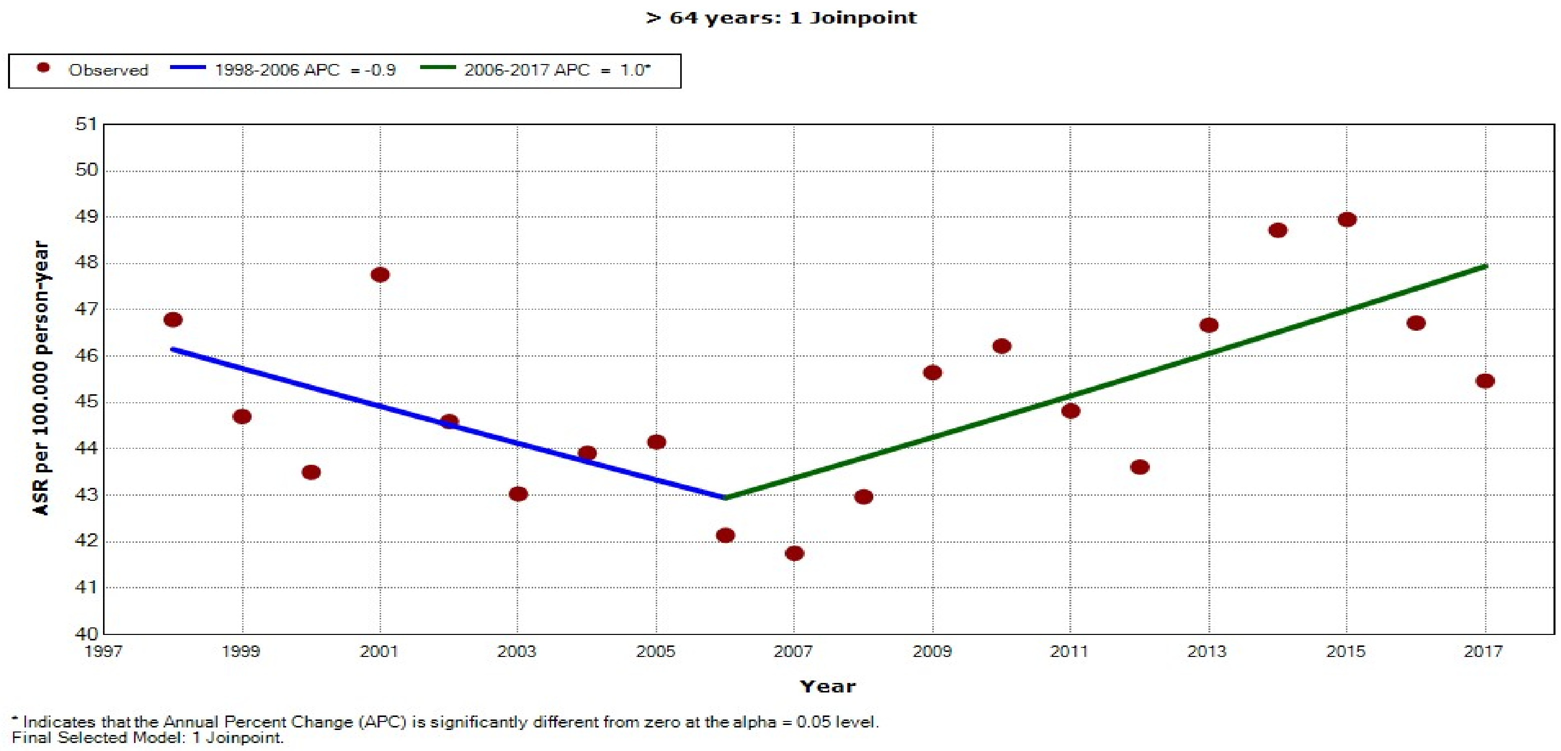

ARS: Annual Standardized rate

Supplementary Figure S5. Age-standardized rate of hospital admissions due to acute appendicitis per 100,000 population, in male aged 65 years or older in Spain, 1998 to 2017.

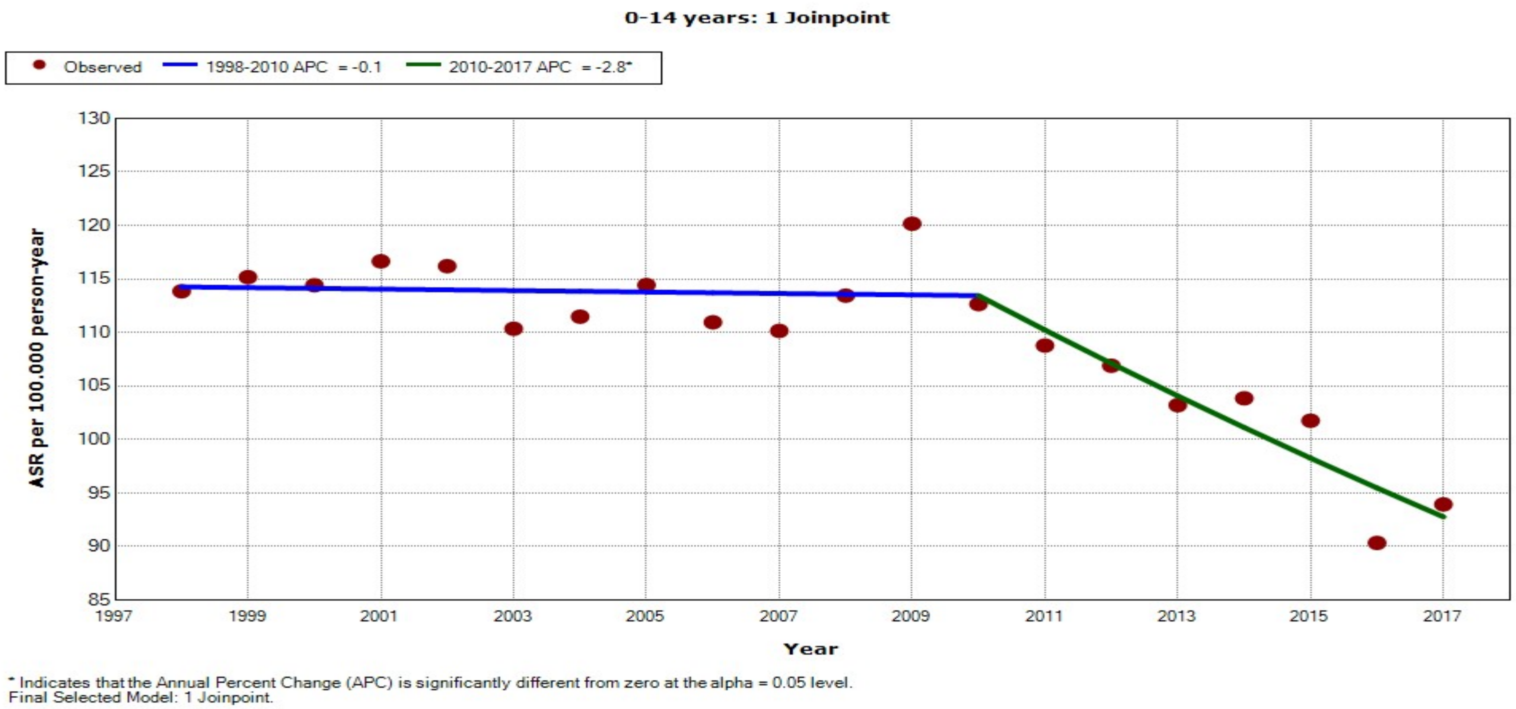

ARS: Annual Standardized rate

Supplementary Figure S6. Age-standardized rate of hospital admissions due to acute appendicitis per 100,000 population, in female aged 0 to 14 years in Spain, 1998 to 2017.

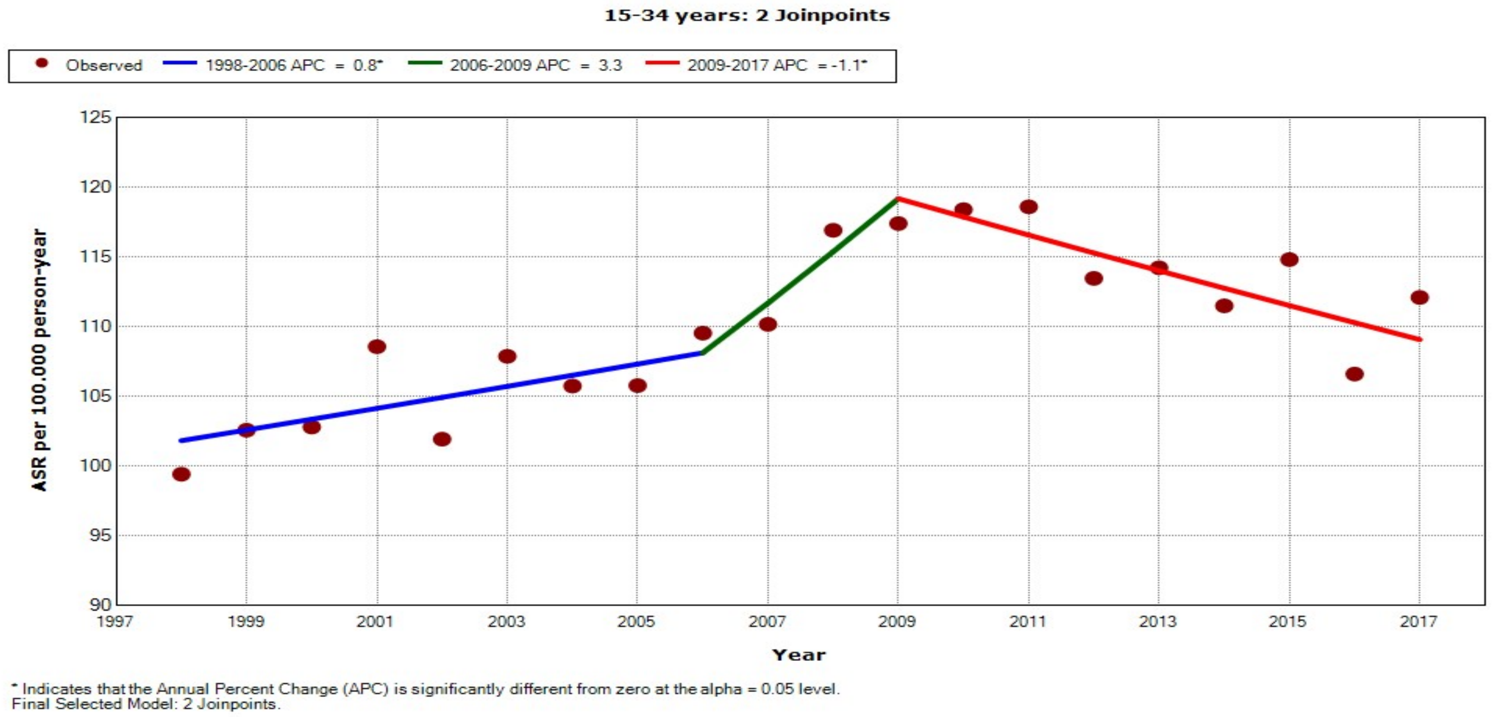

ARS: Annual Standardized rate

Supplementary Figure S7. Age-standardized rate of hospital admissions due to acute appendicitis per 100,000 population, in female aged 15 to 34 years in Spain, 1998 to 2017.

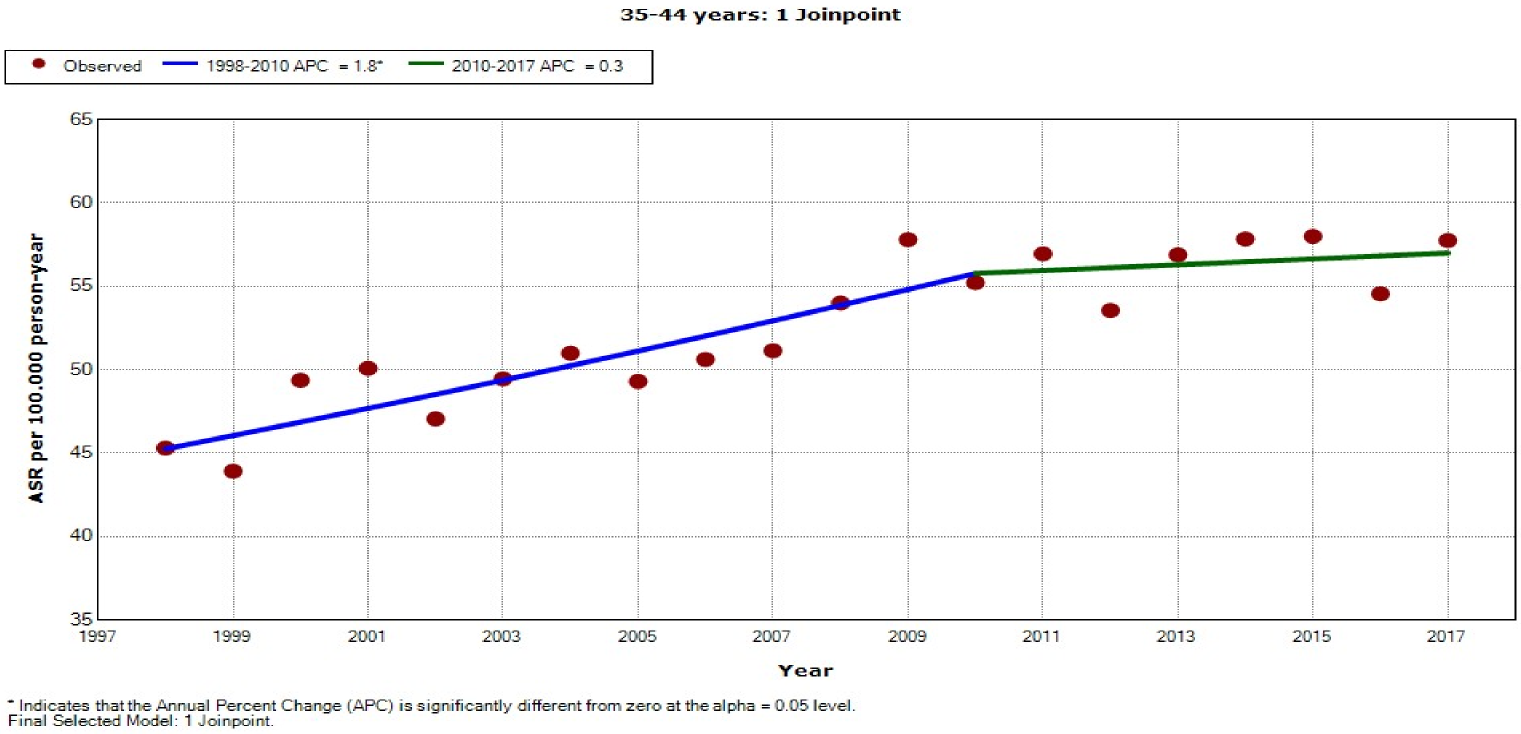

ARS: Annual Standardized rate

Supplementary Figure S8. Age-standardized rate of hospital admissions due to acute appendicitis per 100,000 population, in female aged 35 to 44 years in Spain, 1998 to 2017.

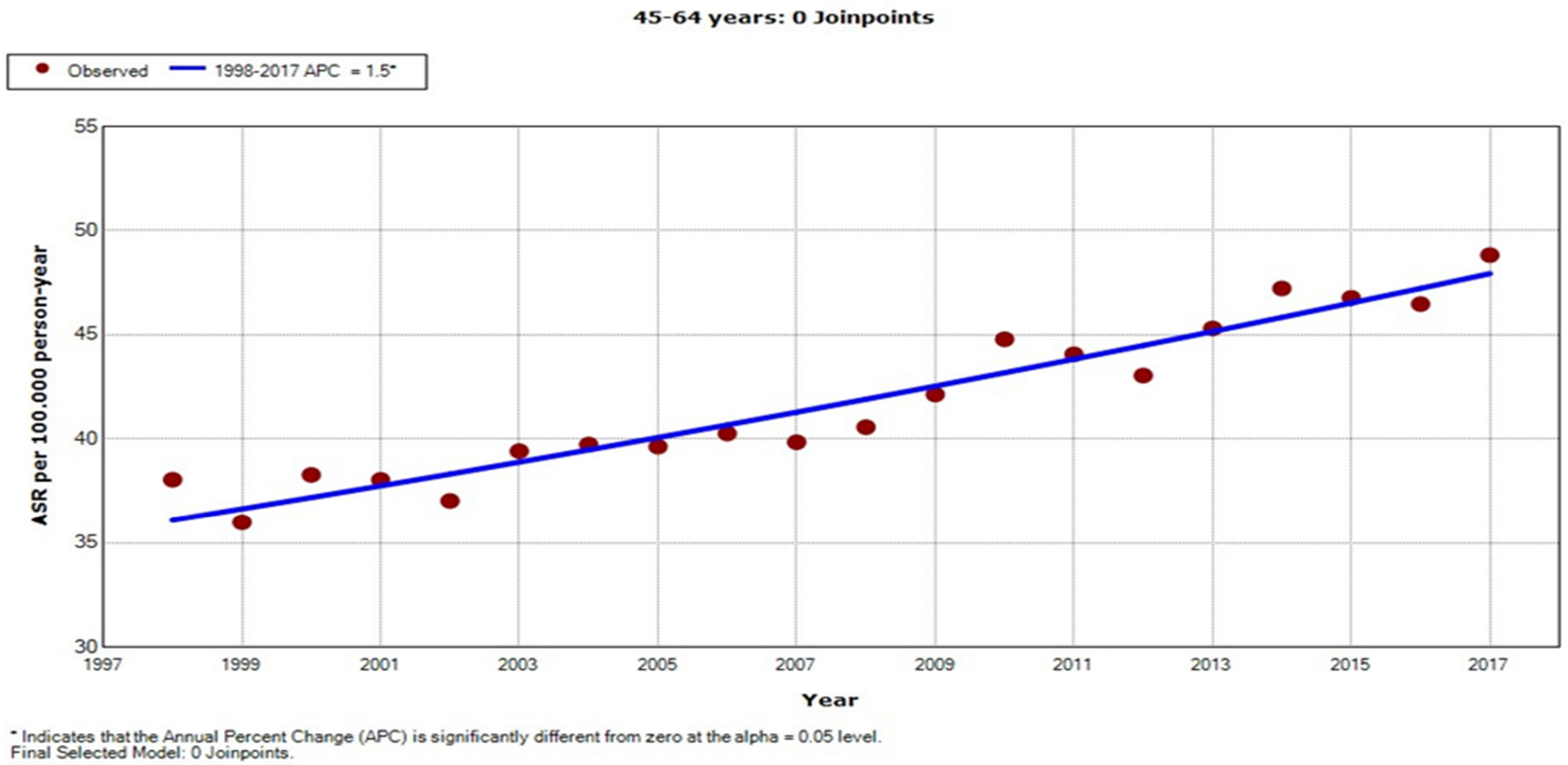

ARS: Annual Standardized rate

Supplementary Figure S9. Age-standardized rate of hospital admissions due to acute appendicitis per 100,000 population, in female aged 45 to 64 years in Spain, 1998 to 2017.

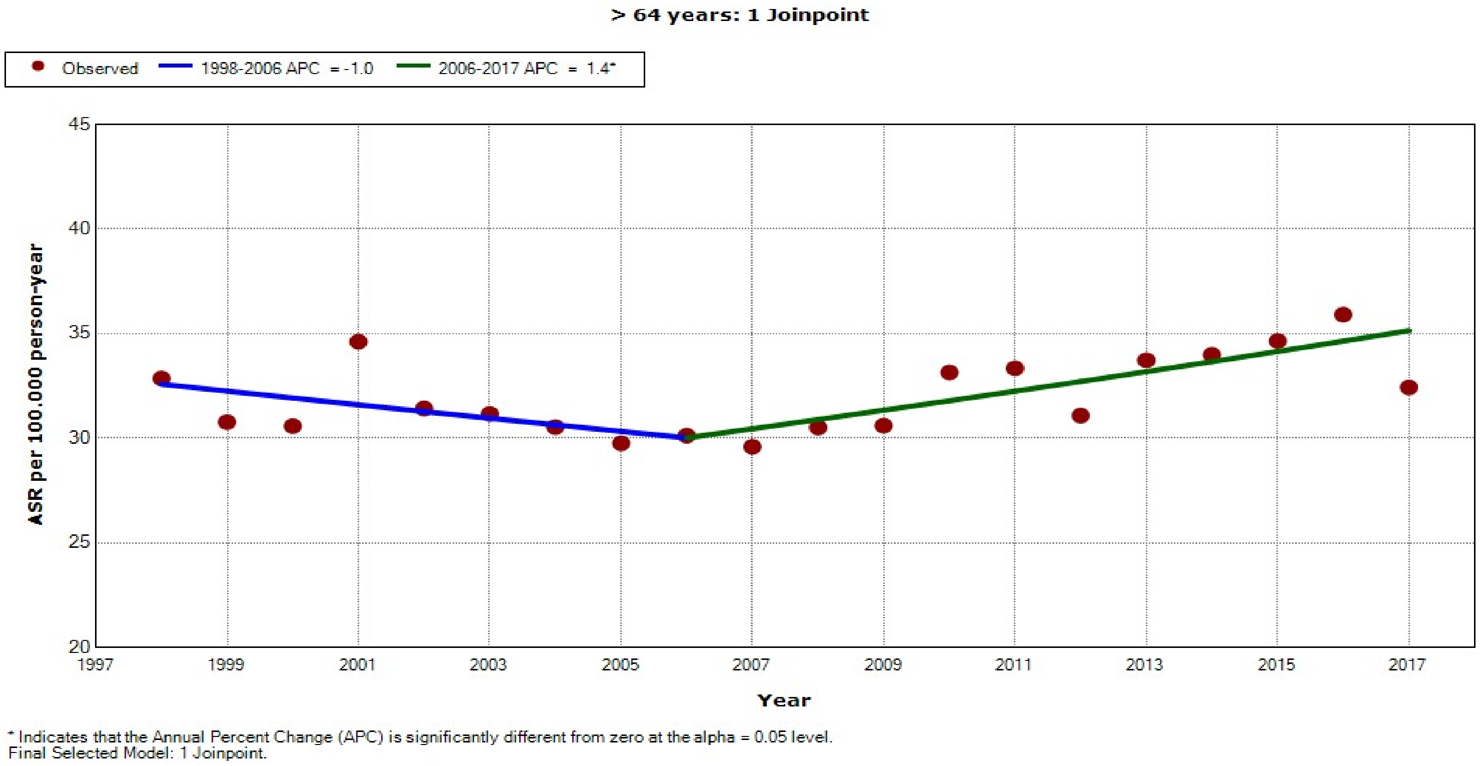

ARS: Annual Standardized rate

Supplementary Figure S10. Age-standardized rate of hospital admissions due to acute appendicitis per 100,000 population, in female aged 65 years or older in Spain, 1998 to 2017.

Supplementary Table S1. Age-standardized rates (ASR) for hospital admissions due to acute appendicitis, by sex and age groups, 1998 to 2017.

		All ages			0-14 years			15-34 years			35-44 years			45-64 years			≥ 65 years		
Gender	Year	n	ASR	95% CI	n	ASR	95% CI	n	ASR	95% CI	n	ASR	95% CI	n	ASR	95% CI	n	ASR	95% CI
Male	1998	20445	95.0	(93.7-96.4)	5709	168.2	(163.8-172.5)	9201	140.3	(137.4-143.1)	1934	67.6	(64.6-70.6)	2314	53.5	(51.3-55.7)	1287	46.8	(44.2-49.4)
	1999	20630	95.7	(94.3-97.0)	5730	171.4	(166.9-175.8)	9288	143.3	(140.4-146.2)	2077	70.6	(67.5-73.6)	2250	51.1	(49.0-53.2)	1285	44.7	(42.2-47.2)
	2000	20917	97.1	(95.7-98.4)	5711	173.9	(169.4-178.5)	9489	147.9	(144.9-150.9)	2162	70.9	(67.9-73.9)	2283	51.4	(49.3-53.6)	1272	43.5	(41.1-45.9)
	2001	21578	99.8	(98.5-101.2)	5731	176.5	(171.9-181.1)	9652	150.6	(147.6-153.6)	2414	75.8	(72.8-78.8)	2348	51.9	(49.8-54.0)	1433	47.8	(45.2-50.3)
	2002	21589	98.4	(97.1-99.8)	5675	174.5	(169.9-179.0)	9823	152.7	(149.6-155.7)	2382	71.9	(69.0-74.8)	2335	50.4	(48.4-52.5)	1374	44.6	(42.2-47.0)
	2003	22101	99.2	(97.9-100.5)	5510	168.0	(163.5-172.4)	10115	156.5	(153.4-159.6)	2584	75.1	(72.2-78.0)	2550	53.5	(51.4-55.6)	1342	43.0	(40.7-45.4)
	2004	22392	100.5	(99.2-101.9)	5752	174.8	(170.3-179.4)	9971	156.0	(152.9-159.1)	2652	75.3	(72.5-78.2)	2645	53.8	(51.8-55.9)	1372	43.9	(41.5-46.3)
	2005	22811	100.9	(99.5-102.2)	5768	174.0	(169.5-178.5)	10201	158.8	(155.7-161.9)	2761	75.9	(73.1-78.7)	2702	52.6	(50.6-54.6)	1379	44.1	(41.8-46.5)
	2006	22531	98.9	(97.5-100.2)	5583	167.4	(163.0-171.8)	10009	157.2	(154.0-160.3)	2849	76.3	(73.5-79.2)	2744	52.0	(50.0-53.9)	1346	42.1	(39.9-44.4)
	2007	22899	100.1	(98.8-101.5)	5957	177.1	(172.6-181.6)	9958	157.6	(154.4-160.7)	2927	76.9	(74.2-79.7)	2710	50.2	(48.3-52.1)	1347	41.8	(39.5-44.0)
	2008	23515	101.3	(100-102.6)	6089	178.0	(173.6-182.5)	10158	159.8	(156.6-162.9)	2995	76.1	(73.4-78.9)	2880	51.6	(49.7-53.5)	1393	43.0	(40.7-45.3)
	2009	24175	103.8	(102.5-105.1)	6298	181.2	(176.8-185.7)	10147	161.6	(158.4-164.8)	3102	77.4	(74.6-80.1)	3117	54.6	(52.7-56.6)	1511	45.7	(43.3-48.0)
	2010	23488	100.9	(99.6-102.2)	5939	168.2	(163.9-172.5)	9526	155.9	(152.8-159.1)	3140	77.5	(74.8-80.2)	3316	56.7	(54.8-58.6)	1567	46.2	(43.9-48.5)
	2011	23436	101.0	(99.6-102.3)	6245	174.2	(169.9-178.5)	9147	154.2	(151.0-157.4)	3192	77.9	(75.2-80.6)	3303	55.4	(53.5-57.3)	1549	44.8	(42.6-47.1)
	2012	22638	98.2	(96.9-99.5)	6309	173.7	(169.4-178.0)	8397	146.7	(143.5-149.9)	3104	75.2	(72.5-77.8)	3296	54.4	(52.5-56.2)	1532	43.6	(41.4-45.8)
	2013	22505	98.5	(97.2-99.8)	6293	171.6	(167.3-175.8)	8191	148.3	(145.0-151.5)	3031	73.6	(71-76.2.0)	3328	54.0	(52.2-55.9)	1662	46.7	(44.4-48.9)
	2014	22379	98.8	(97.5-100.1)	6308	170.5	(166.3-174.7)	7755	146.0	(142.7-149.2)	3163	77.4	(74.7-80.1)	3382	54.4	(52.6-56.3)	1771	48.7	(46.4-51.0)
	2015	22132	98.2	(96.9-99.5)	6125	164.0	(159.9-168.1)	7632	147.3	(144.0-150.6)	3139	77.8	(75.1-80.5)	3434	54.7	(52.8-56.5)	1802	49.0	(46.7-51.2)
	2016	20820	92.5	(91.2-93.7)	5630	150.0	(146.1-153.9)	7071	138.5	(135.3-141.8)	2931	73.5	(70.9-76.2)	3453	54.0	(52.2-55.8)	1735	46.7	(44.5-48.9)
	2017	21329	94.7	(93.4-95.9)	5780	153.0	(149.1-157.0)	7235	143.4	(140.1-146.7)	3026	77.2	(74.5-80.0)	3571	54.9	(53.1-56.8)	1717	45.5	(43.3-47.6)
Female	1998	14201	66.0	(64.9-67.1)	3667	113.9	(110.1-117.6)	6283	99.4	(96.9-101.9)	1298	45.3	(42.8-47.8)	1710	38.0	(36.2-39.9)	1243	32.9	(31.0-34.7)
	1999	14132	65.9	(64.8-67.0)	3614	115.2	(111.4-118.9)	6384	102.6	(100.0-105.1)	1291	43.9	(41.5-46.3)	1640	36.0	(34.2-37.7)	1203	30.8	(29.0-32.5)
	2000	14360	67.1	(66.0-68.2)	3547	114.4	(110.6-118.2)	6334	102.8	(100.2-105.3)	1504	49.3	(46.8-51.8)	1759	38.3	(36.5-40.1)	1216	30.6	(28.8-32.3)
	2001	14976	69.7	(68.6-70.9)	3566	116.7	(112.8-120.5)	6652	108.6	(105.9-111.2)	1575	50.1	(47.6-52.5)	1774	38.0	(36.3-39.8)	1409	34.6	(32.8-36.4)
	2002	14365	66.7	(65.6-67.8)	3552	116.2	(112.4-120.0)	6222	101.9	(99.4-104.5)	1530	47.0	(44.7-49.4)	1758	37.0	(35.3-38.8)	1303	31.4	(29.7-33.1)
	2003	14913	68.1	(67.0-69.2)	3419	110.4	(106.6-114.1)	6596	107.9	(105.2-110.5)	1659	49.4	(47.1-51.8)	1928	39.4	(37.6-41.2)	1311	31.2	(29.5-32.8)
	2004	14875	67.9	(66.8-69.1)	3455	111.5	(107.8-115.2)	6400	105.7	(103.1-108.3)	1739	51.0	(48.6-53.4)	1994	39.7	(38.0-41.5)	1287	30.5	(28.8-32.2)
	2005	15037	68.0	(66.9-69.1)	3579	114.4	(110.7-118.2)	6399	105.8	(103.1-108.4)	1728	49.3	(47.0-51.6)	2072	39.6	(37.9-41.3)	1259	29.8	(28.1-31.4)
	2006	15333	68.8	(67.7-69.9)	3491	111.0	(107.3-114.6)	6582	109.5	(106.8-112.2)	1804	50.6	(48.3-53.0)	2158	40.3	(38.6-42.0)	1298	30.1	(28.5-31.8)
	2007	15375	68.6	(67.5-69.7)	3503	110.1	(106.5-113.8)	6548	110.1	(107.4-112.8)	1855	51.1	(48.8-53.5)	2191	39.8	(38.2-41.5)	1278	29.6	(27.9-31.2)
	2008	16295	71.6	(70.5-72.7)	3670	113.4	(109.8-117.1)	6984	116.9	(114.1-119.7)	2012	54.0	(51.6-56.4)	2303	40.6	(38.9-42.2)	1326	30.5	(28.8-32.2)
	2009	16905	73.8	(72.7-74.9)	3954	120.2	(116.4-123.9)	6957	117.4	(114.6-120.2)	2192	57.8	(55.4-60.2)	2448	42.1	(40.5-43.8)	1354	30.6	(28.9-32.2)
	2010	16886	73.6	(72.5-74.7)	3766	112.7	(109.1-116.3)	6856	118.4	(115.5-121.2)	2118	55.2	(52.9-57.6)	2658	44.8	(43.1-46.5)	1488	33.1	(31.4-34.8)
	2011	16795	73.1	(72.0-74.2)	3691	108.8	(105.3-112.3)	6710	118.6	(115.7-121.5)	2208	56.9	(54.6-59.3)	2658	44.1	(42.4-45.7)	1528	33.3	(31.6-35.0)
	2012	16085	70.4	(69.3-71.5)	3672	106.9	(103.4-110.4)	6222	113.4	(110.6-116.3)	2095	53.5	(51.3-55.8)	2655	43.0	(41.4-44.7)	1441	31.1	(29.4-32.7)
	2013	16292	71.5	(70.4-72.6)	3588	103.2	(99.8-106.6)	6054	114.2	(111.3-117.1)	2228	56.9	(54.5-59.2)	2840	45.3	(43.6-47.0)	1582	33.7	(32.0-35.4)
	2014	16232	71.6	(70.5-72.8)	3647	103.9	(100.5-107.2)	5745	111.5	(108.6-114.4)	2254	57.8	(55.4-60.2)	2980	47.2	(45.5-48.9)	1606	34.0	(32.3-35.7)
	2015	16254	72.1	(71.0-73.2)	3595	101.8	(98.4-105.1)	5761	114.8	(111.8-117.8)	2244	58.0	(55.6-60.4)	2993	46.8	(45.1-48.4)	1661	34.6	(32.9-36.3)
	2016	15333	67.9	(66.8-69.0)	3203	90.3	(87.2-93.5)	5270	106.6	(103.7-109.5)	2094	54.5	(52.2-56.9)	3019	46.5	(44.8-48.1)	1747	35.9	(34.2-37.6)

		All ages			0-14 years			15-34 years			35-44 years			45-64 years			≥ 65 years		
Gender	Year	n	ASR	95% CI	n	ASR	95% CI	n	ASR	95% CI	n	ASR	95% CI	n	ASR	95% CI	n	ASR	95% CI
	2017	15823	70.2	(69.1-71.3)	3350	93.9	(90.8-97.1)	5475	112.1	(109.1-115.1)	2192	57.7	(55.3-60.2)	3216	48.8	(47.1-50.5)	1590	32.4	(30.8-34.0)

Supplementary Table S2. Mean length of stay (days) due to acute appendicitis, by sex and age group, 1998 to 2017.

		All			0-14 years			15-34 years			35-44 years			45-64 years			> 64 years		
**Gender**	**Year**	**n**	**Mean**	**95% CI**	**n**	**Mean**	**95% CI**	**n**	**Mean**	**95% CI**	**n**	**Mean**	**95% CI**	**n**	**Mean**	**95% CI**	**n**	**Mean**	**95% CI**
Male	1998	19200	4.6	(4.6-4.6)	5477	4.7	(4.6-4.7)	8959	4.0	(4.0-4.1)	1808	4.8	(4.7-4.9)	2034	5.7	(5.6-5.8)	922	6.8	(6.6-6.9)
	1999	19464	4.5	(4.4-4.5)	5540	4.5	(4.4-4.5)	9021	3.9	(3.9-4.0)	1954	4.7	(4.6-4.8)	1979	5.5	(5.4-5.7)	970	6.6	(6.5-6.8)
	2000	19654	4.4	(4.4-4.5)	5466	4.5	(4.5-4.6)	9213	3.8	(3.8-3.9)	2037	4.5	(4.4-4.7)	1990	5.4	(5.3-5.6)	948	6.7	(6.5-6.9)
	2001	20298	4.3	(4.3-4.4)	5471	4.5	(4.4-4.6)	9411	3.8	(3.7-3.8)	2279	4.4	(4.3-4.5)	2061	5.3	(5.2-5.4)	1076	6.4	(6.2-6.6)
	2002	20344	4.3	(4.2-4.3)	5434	4.4	(4.4-4.5)	9567	3.7	(3.6-3.7)	2262	4.5	(4.4-4.6)	2066	5.2	(5.1-5.4)	1015	6.5	(6.3-6.6)
	2003	20850	4.2	(4.1-4.2)	5279	4.4	(4.3-4.5)	9841	3.6	(3.6-3.6)	2422	4.2	(4.1-4.3)	2287	5.1	(5.0-5.2)	1021	6.4	(6.3-6.6)
	2004	21150	4.2	(4.1-4.2)	5511	4.3	(4.2-4.4)	9715	3.5	(3.5-3.6)	2529	4.3	(4.2-4.4)	2359	5.1	(5.0-5.2)	1036	6.5	(6.3-6.7)
	2005	21620	4.1	(4.0-4.1)	5540	4.3	(4.2-4.4)	9943	3.5	(3.4-3.5)	2643	4.2	(4.1-4.3)	2448	5.0	(4.9-5.1)	1046	6.3	(6.1-6.5)
	2006	21392	4.0	(4.0-4.0)	5349	4.2	(4.2-4.3)	9769	3.4	(3.4-3.5)	2732	4.0	(3.9-4.1)	2506	4.8	(4.7-4.9)	1036	6.2	(6.0-6.4)
	2007	21727	3.9	(3.9-3.9)	5733	4.1	(4.1-4.2)	9732	3.3	(3.3-3.3)	2784	4.0	(3.9-4.0)	2450	4.8	(4.7-4.9)	1028	6.0	(5.8-6.2)
	2008	22424	3.8	(3.8-3.9)	5892	4.1	(4.0-4.2)	9929	3.2	(3.2-3.3)	2882	3.9	(3.8-4.0)	2632	4.7	(4.6-4.8)	1089	5.9	(5.7-6.0)
	2009	23143	3.7	(3.7-3.8)	6064	4.0	(4.0-4.1)	9980	3.1	(3.1-3.2)	2997	3.7	(3.6-3.8)	2890	4.4	(4.3-4.5)	1212	5.8	(5.6-6.0)
	2010	22476	3.6	(3.6-3.6)	5731	3.8	(3.8-3.9)	9353	3.0	(3.0-3.1)	3037	3.5	(3.4-3.6)	3068	4.3	(4.2-4.4)	1287	5.6	(5.4-5.7)
	2011	22523	3.5	(3.5-3.6)	6034	3.8	(3.7-3.8)	8975	2.9	(2.9-2.9)	3108	3.4	(3.4-3.5)	3093	4.2	(4.1-4.3)	1313	5.4	(5.2-5.5)
	2012	21753	3.5	(3.5-3.5)	6087	3.7	(3.6-3.7)	8269	2.9	(2.8-2.9)	3000	3.4	(3.3-3.4)	3094	4.1	(4.0-4.2)	1303	5.5	(5.3-5.6)
	2013	21633	3.4	(3.4-3.5)	6076	3.6	(3.6-3.7)	8056	2.8	(2.7-2.8)	2940	3.3	(3.2-3.4)	3144	4.1	(4.0-4.2)	1417	5.3	(5.1-5.4)
	2014	21540	3.5	(3.4-3.5)	6103	3.6	(3.5-3.7)	7633	2.8	(2.7-2.8)	3087	3.3	(3.2-3.3)	3196	4.0	(4.0-4.1)	1521	5.4	(5.3-5.5)
	2015	21295	3.4	(3.4-3.4)	5918	3.5	(3.5-3.6)	7503	2.7	(2.7-2.8)	3061	3.2	(3.2-3.3)	3254	4.0	(3.9-4.0)	1559	5.1	(5.0-5.3)
	2016	20051	3.5	(3.5-3.5)	5439	3.6	(3.5-3.6)	6946	2.9	(2.8-2.9)	2870	3.3	(3.2-3.4)	3287	4.0	(3.9-4.1)	1509	5.2	(5.1-5.3)
	2017	20597	3.4	(3.3-3.4)	5608	3.5	(3.5-3.6)	7126	2.7	(2.7-2.8)	2957	3.2	(3.1-3.2)	3410	3.9	(3.8-4.0)	1496	5.0	(4.8-5.1)
Female	1998	13416	4.5	(4.4-4.5)	3511	4.6	(4.6-4.7)	6186	3.7	(3.7-3.8)	1258	4.5	(4.3-4.6)	1513	5.5	(5.3-5.6)	948	6.7	(6.6-6.9)
	1999	13398	4.4	(4.3-4.4)	3469	4.6	(4.6-4.7)	6257	3.6	(3.6-3.7)	1240	4.4	(4.2-4.5)	1479	5.3	(5.1-5.4)	953	6.5	(6.3-6.7)
	2000	13576	4.3	(4.3-4.4)	3380	4.6	(4.5-4.6)	6212	3.6	(3.5-3.6)	1439	4.4	(4.2-4.5)	1582	5.2	(5.1-5.3)	963	6.7	(6.5-6.8)
	2001	14180	4.3	(4.2-4.3)	3410	4.6	(4.5-4.7)	6538	3.6	(3.5-3.6)	1523	4.1	(4.0-4.2)	1601	5.1	(4.9-5.2)	1108	6.3	(6.1-6.4)
	2002	13559	4.2	(4.1-4.2)	3372	4.5	(4.4-4.6)	6103	3.5	(3.4-3.5)	1468	4.1	(4.0-4.2)	1610	4.8	(4.7-4.9)	1006	6.1	(5.9-6.3)
	2003	14041	4.1	(4.1-4.1)	3253	4.5	(4.4-4.6)	6433	3.4	(3.4-3.5)	1587	3.9	(3.8-4.0)	1758	4.9	(4.8-5.0)	1010	6.3	(6.1-6.5)
	2004	14093	4.0	(4.0-4.1)	3314	4.3	(4.2-4.4)	6256	3.4	(3.4-3.4)	1673	3.8	(3.7-3.9)	1832	4.8	(4.7-4.9)	1018	6.1	(6.0-6.3)
	2005	14250	4.0	(3.9-4.0)	3427	4.3	(4.2-4.4)	6258	3.3	(3.3-3.3)	1656	3.9	(3.8-4.0)	1925	4.6	(4.5-4.7)	984	6.1	(5.9-6.3)
	2006	14607	3.9	(3.9-4.0)	3350	4.3	(4.3-4.4)	6469	3.2	(3.2-3.3)	1736	3.8	(3.7-3.9)	2000	4.4	(4.3-4.5)	1052	6.1	(5.9-6.2)
	2007	14705	3.9	(3.8-3.9)	3372	4.2	(4.1-4.3)	6435	3.2	(3.2-3.3)	1796	3.7	(3.6-3.8)	2029	4.4	(4.3-4.5)	1073	6.0	(5.8-6.2)
	2008	15659	3.8	(3.7-3.8)	3522	4.1	(4.0-4.2)	6888	3.1	(3.1-3.1)	1955	3.5	(3.4-3.6)	2183	4.4	(4.3-4.5)	1111	5.8	(5.6-6.0)
	2009	16288	3.6	(3.6-3.6)	3812	4.0	(3.9-4.0)	6862	2.9	(2.9-3.0)	2143	3.4	(3.3-3.5)	2306	4.1	(4.0-4.2)	1165	5.6	(5.5-5.8)
	2010	16231	3.5	(3.5-3.6)	3605	3.9	(3.9-4.0)	6764	2.9	(2.8-2.9)	2074	3.2	(3.1-3.3)	2519	4.0	(3.9-4.1)	1269	5.5	(5.4-5.7)
	2011	16225	3.4	(3.4-3.5)	3553	3.8	(3.8-3.9)	6632	2.7	(2.7-2.8)	2146	3.1	(3.1-3.2)	2553	3.9	(3.8-4.0)	1341	5.3	(5.1-5.4)
	2012	15524	3.4	(3.4-3.5)	3529	3.8	(3.7-3.9)	6156	2.8	(2.7-2.8)	2049	3.1	(3.0-3.2)	2532	3.8	(3.8-3.9)	1258	5.2	(5.1-5.4)
	2013	15738	3.4	(3.3-3.4)	3452	3.8	(3.7-3.8)	5991	2.6	(2.6-2.7)	2169	3.0	(3.0-3.1)	2726	3.8	(3.7-3.9)	1400	5.2	(5.0-5.3)
	2014	15677	3.4	(3.4-3.4)	3520	3.8	(3.7-3.8)	5680	2.7	(2.6-2.7)	2202	3.1	(3.0-3.2)	2855	3.7	(3.6-3.8)	1420	5.2	(5.0-5.3)
	2015	15754	3.3	(3.3-3.4)	3470	3.6	(3.5-3.7)	5694	2.7	(2.6-2.7)	2205	3.0	(2.9-3.0)	2894	3.7	(3.6-3.8)	1491	5.1	(4.9-5.2)

		All			0-14 years			15-34 years			35-44 years			45-64 years			> 64 years		
**Gender**	**Year**	**n**	**Mean**	**95% CI**	**n**	**Mean**	**95% CI**	**n**	**Mean**	**95% CI**	**n**	**Mean**	**95% CI**	**n**	**Mean**	**95% CI**	**n**	**Mean**	**95% CI**
	2016	14760	3.4	(3.4-3.5)	3062	3.6	(3.5-3.7)	5217	2.8	(2.7-2.8)	2053	3.1	(3.0-3.2)	2885	3.8	(3.7-3.9)	1543	5.1	(5.0-5.3)
	2017	15311	3.3	(3.3-3.3)	3221	3.7	(3.6-3.7)	5424	2.6	(2.6-2.7)	2158	3.0	(2.9-3.1)	3104	3.5	(3.5-3.6)	1404	5.0	(4.8-5.1)
